# Modified rotational wedge distal metatarsal osteotomy versus chevron osteotomy for hallux valgus: long-term radiographic and clinical outcomes

**DOI:** 10.1007/s00402-026-06315-2

**Published:** 2026-04-17

**Authors:** Ahmet Aybar, Kemal Gokkus, Erdem Özden, Mehmet Hamdi Çetin, Mehmet Ümit Çetin

**Affiliations:** 1 Department of Orthopedics and Traumatology, University of Health Sciences, Gaziosmanpasa Training and Research Hospital, Istanbul, Turkey; 2 Department of Orthopedics and Traumatology, Alanya Research and Practice Center, Baskent University School of Medicine, Antalya, Turkey; 3 Department of Orthopedics and Traumatology, Faculty of Medicine, Tekirdag Namik Kemal University, Tekirdag, Turkey

**Keywords:** Hallux valgus, Distal metatarsal osteotomy, Sesamoid position, Radiographic recurrence

## Abstract

**Background:**

Distal Chevron osteotomy is commonly used for mild-to-moderate hallux valgus, but long-term loss of correction and radiographic recurrence remain concerns, particularly when distal articular alignment and sesamoid position are not adequately restored. We compared long-term radiographic and clinical outcomes of a modified rotational wedge distal metatarsal osteotomy versus standard distal Chevron osteotomy in adults with mild-to-moderate symptomatic hallux valgus.

**Methods:**

In this single-center retrospective cohort study, 100 feet (100 patients) treated between 2010 and 2019 were analyzed (Modified, *n* = 46; Chevron, *n* = 54) at a mean follow-up of 101.2 ± 11.5 months. Soft-tissue balancing was standardized, with an intra-articular lateral release performed in both groups. Outcomes included radiographic measures (hallux valgus angle [HVA], intermetatarsal angle [IMA], distal metatarsal articular angle [DMAA], and medial sesamoid position), clinical scores (AOFAS, VAS), recurrence, and complications. Radiographic recurrence was defined as final HVA > 15°.

**Results:**

Final AOFAS scores were similar between groups (*p* = 0.621), and the between-group difference in final VAS pain scores did not remain significant after Benjamini-Hochberg false discovery rate (BH-FDR) adjustment (q = 0.057). Compared with the Chevron group, the Modified group demonstrated superior final radiographic alignment, with lower HVA and IMA (both *p* < 0.001) and lower DMAA (*p* = 0.002). Despite worse baseline sesamoid subluxation, the Modified group achieved a more central final sesamoid position (*p* = 0.010). Radiographic recurrence was less frequent in the Modified group (4.3% vs. 27.8%), representing a relative risk of 0.16 (95% CI 0.04–0.65; *p* = 0.003); this association persisted after inverse probability of treatment weighting (adjusted odds ratio [aOR] 0.09, 95% CI 0.01–0.60; *p* = 0.013). Complication rates were low and comparable.

**Conclusions:**

At long-term follow-up, the modified rotational wedge distal osteotomy yielded superior radiographic alignment and a lower recurrence rate than distal Chevron osteotomy, without higher complication rates, while functional outcomes were similar.

**Supplementary Information:**

The online version contains supplementary material available at 10.1007/s00402-026-06315-2.

## Introduction

 Hallux valgus is one of the most common forefoot deformities in adults, particularly in women, and is associated with pain, footwear intolerance, and activity limitation [1–3]. When symptoms persist despite nonoperative measures, distal metatarsal osteotomies are widely used for correction of mild-to-moderate deformity [2]. 

Distal Chevron osteotomy is an established, extensively studied technique that provides reliable short- to mid-term correction and functional improvement in appropriately selected patients [4, 5]. Nevertheless, several studies have shown progressive loss of correction and clinically relevant recurrence over time, especially in patients with larger preoperative deformities or inadequate correction of the distal metatarsal articular angle (DMAA) and sesamoid position [6–8]. 

These concerns have contributed to a growing recognition of hallux valgus as a three-dimensional deformity, in which coronal rotation of the first metatarsal and sesamoid malalignment are clinically relevant components [9, 10]. Accordingly, long-term surgical durability may depend not only on correction of angular deformity but also on the quality of triplanar correction, including restoration of sesamoid position; however, the evidence on this point is not uniform across studies [8, 11, 12]. 

A modified distal metatarsal osteotomy described by Çetin et al. was designed to correct multiple pathologic elements of hallux valgus (hallux valgus angle [HVA], intermetatarsal angle [IMA], DMAA, and sesamoid displacement) using a guided wedge-based osteotomy [13]. However, long-term comparative evidence regarding whether this technique provides more durable radiographic correction and lower recurrence than the conventional distal Chevron osteotomy remains limited.

Therefore, the aim of this study was to compare long-term radiographic and clinical outcomes of the modified distal metatarsal osteotomy versus the standard distal Chevron osteotomy in adults with mild-to-moderate symptomatic hallux valgus. We hypothesized that the modified technique would achieve greater and more durable improvements in HVA, IMA, DMAA, and medial sesamoid position, resulting in a lower rate of radiographic recurrence, while providing comparable pain relief, functional scores, and complication rates.

## Materials and methods

### Study design and participants

This single-center, retrospective comparative cohort study was conducted at a tertiary referral hospital. The protocol was approved by the Clinical Research Ethics Committee of Gaziosmanpasa Training and Research Hospital (Decision No. 86, dated 25 May 2022) and complied with the Declaration of Helsinki. Hospital records were reviewed to identify all consecutive patients who underwent surgical correction for symptomatic hallux valgus between January 2010 and December 2019. Patients who met eligibility criteria and were included in the final cohort attended a dedicated follow-up visit that included clinical assessment and standardized weight-bearing radiographs; written informed consent was obtained at that visit.

### Inclusion and exclusion criteria

Inclusion criteria were: (1) age ≥ 18 years; (2) a diagnosis of symptomatic hallux valgus refractory to a minimum of 6 months of conservative management; and (3) a minimum clinical and radiographic follow-up of 60 months. Exclusion criteria were applied to promote cohort comparability and minimize confounding: (1) severe deformity, defined as a preoperative hallux valgus angle (HVA) > 40° or intermetatarsal angle (IMA) > 20°; (2) presence of inflammatory arthropathy (e.g., rheumatoid arthritis), neuromuscular disorders, or previous surgery on the index foot; and (3) performance of concomitant procedures for correction of lesser toe deformities during the index surgery. No additional proximal phalangeal osteotomy (such as an Akin procedure) or other first-ray procedure was performed in any patient; deformity correction relied solely on the distal first metatarsal osteotomy and soft-tissue balancing.

### Study groups

From an initial pool of 114 consecutive patients screened, 14 were excluded based on the prespecified eligibility criteria, primarily due to insufficient follow-up or severe deformity, yielding a final study population of 100 patients (100 feet). This cohort was divided into two groups according to the surgical technique performed:

- Modified Group (*n* = 46): Patients treated with the modified rotational wedge distal metatarsal osteotomy described by Çetin et al., characterized by a guided trapezoidal wedge cut with a rotational component and fixation with a headless compression screw. Soft-tissue balancing, including an intra-articular lateral release, was standardized across both cohorts.
- Chevron Group (*n* = 54): Patients treated with a conventional distal Chevron osteotomy. To standardize soft-tissue balancing between groups, an intra-articular lateral release was performed in all cases (Surgical Technique).

### Surgical technique

All operative procedures were performed in a standardized operating room environment by one of two senior fellowship-trained foot and ankle surgeons. The choice of anesthesia (spinal or general) was determined by preoperative anesthesiology assessment. A single preoperative dose of intravenous cefazolin (1 g) was administered for antibiotic prophylaxis. No pneumatic tourniquet was utilized in either group, to allow continuous intraoperative assessment of capillary perfusion.

#### Modified group surgical procedure

**Fig. 1:**
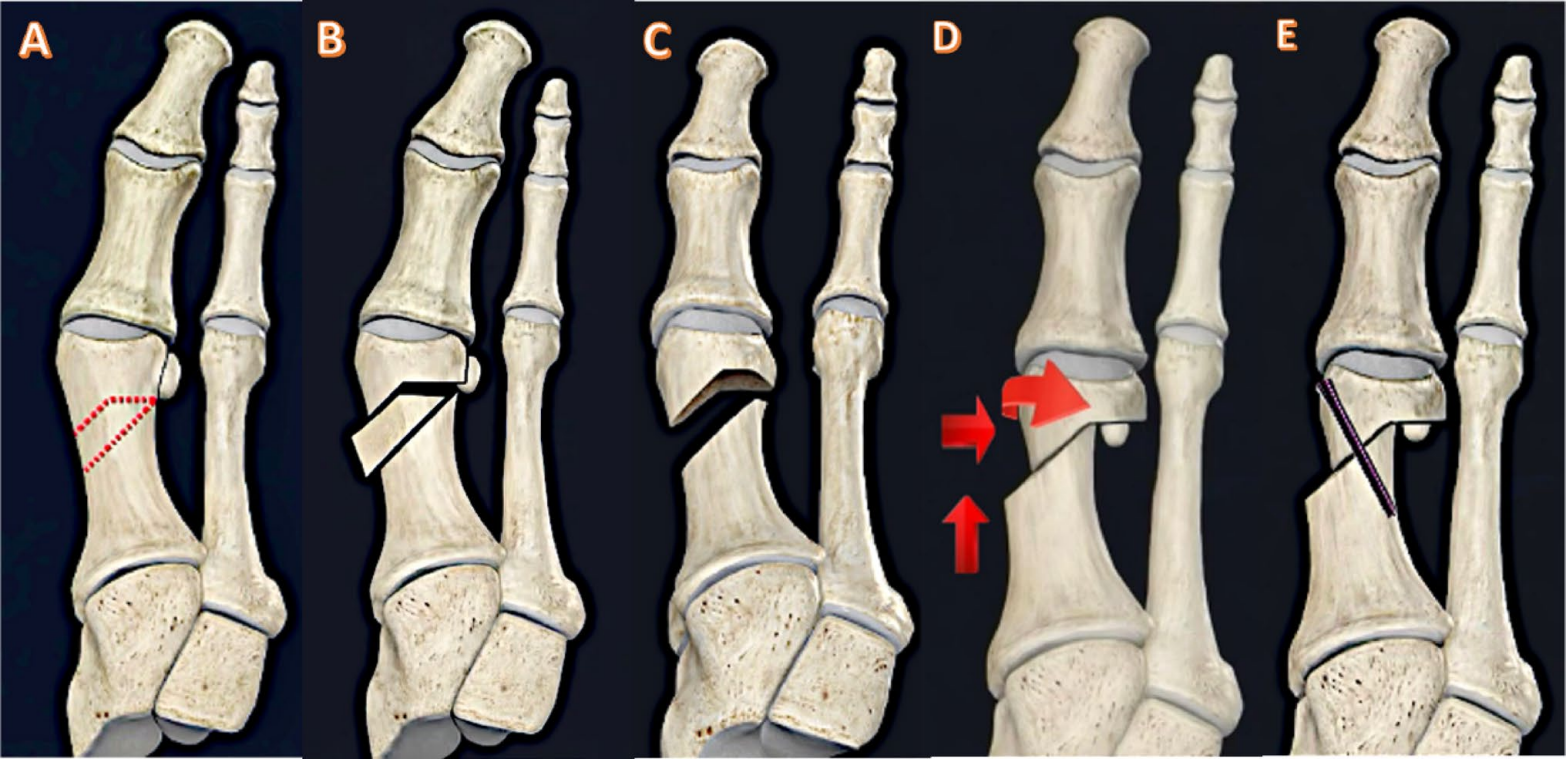
Stepwise schematic of the modified rotational wedge distal first metatarsal osteotomy (Modified group). **A** Planned wedge geometry at the distal first metatarsal. **B** Excision of the trapezoidal wedge fragment. **C** Wedge removal. **D** Controlled reduction to achieve realignment (arrows indicate direction of correction). **E** Final fixation with a headless compression screw

**Fig. 2:**
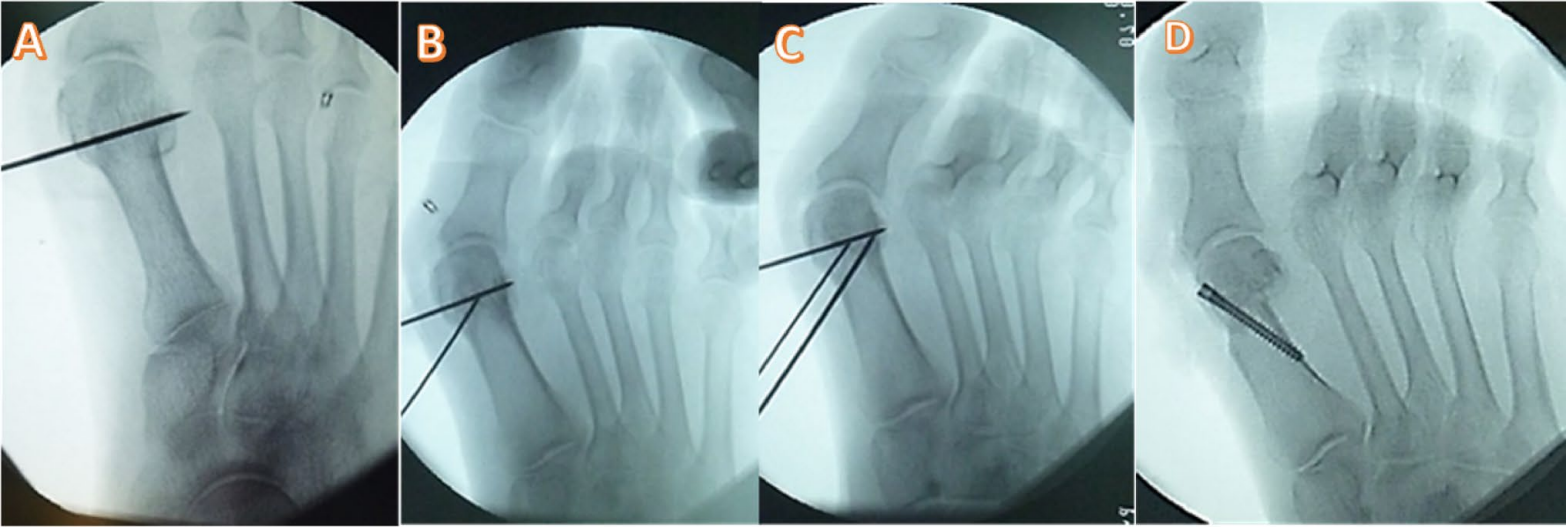
Intraoperative fluoroscopic images of the modified rotational wedge osteotomy (Modified group). **A** Initial K-wire placement under fluoroscopic guidance. **B** K-wire-guided delineation and completion of the wedge osteotomy. **C** Reduction of the capital fragment to restore alignment. **D** Final fixation with a headless compression screw

**Fig. 3:**
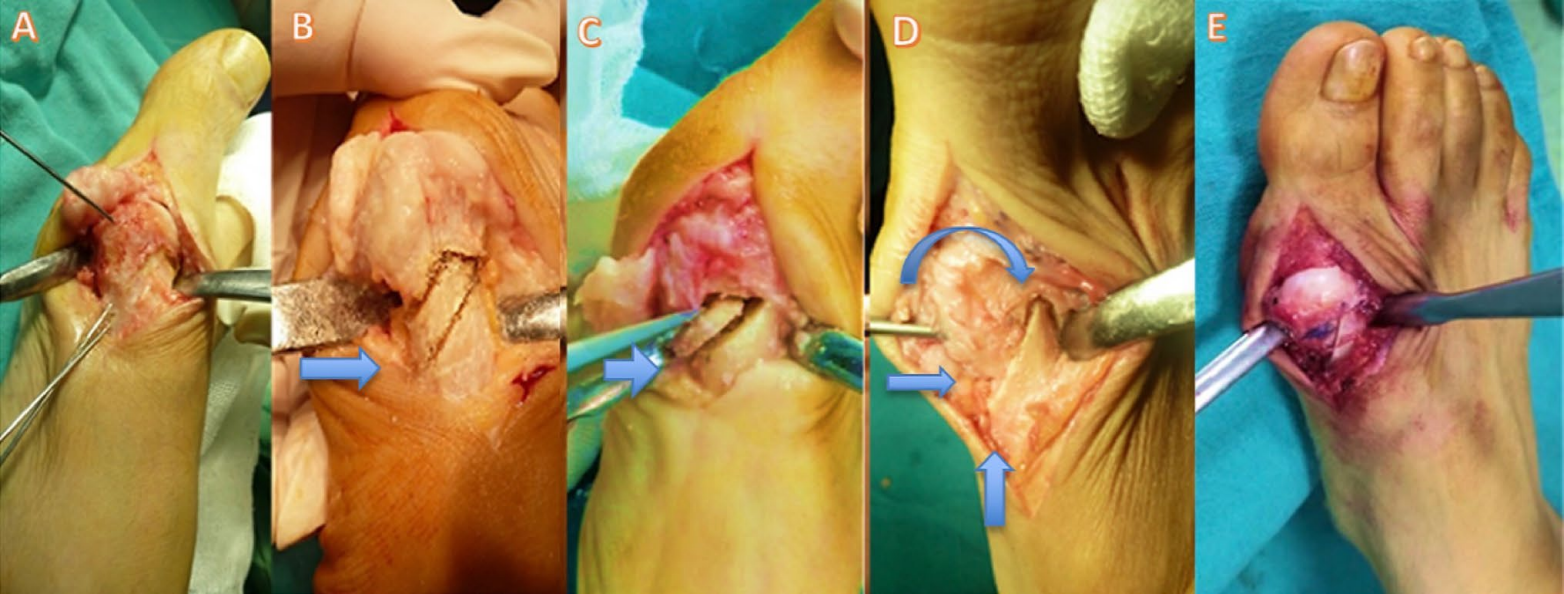
Intraoperative photographs of the modified rotational wedge osteotomy (Modified group). **A** Dorsomedial surgical exposure of the first metatarsophalangeal joint. **B** Execution of the wedge osteotomy (arrow indicates the osteotomy plane). **C** Extraction of the trapezoidal wedge fragment (arrow). **D** Multiplanar reduction of the capital fragment: arrows indicate the directions of lateral translation (horizontal arrow), coronal derotation (curved arrow), and angular correction (vertical arrow). **E** Intraoperative appearance after reduction, demonstrating restored alignment of the first ray prior to closure

The technique followed the principles detailed by Çetin et al. [13] (Fig. 1). A dorsomedial longitudinal incision was made. A formal intra-articular lateral release was systematically performed, encompassing the lateral joint capsule, the conjoined tendon of the adductor hallucis, and the lateral sesamoid ligament to achieve complete mobilization of the sesamoid complex. Under fluoroscopic guidance, a medial-based trapezoidal wedge osteotomy was precisely planned using three K-wires as guides (Fig. 2). Following wedge removal, the capital fragment was repositioned to achieve the planned multiplanar correction (lateral translation and manual intraoperative coronal derotation, guided by fluoroscopic assessment of sesamoid position) and definitively fixed with a headless compression screw (Fig. 3). A medial capsulorrhaphy completed the procedure. No Akin or other proximal phalangeal osteotomy was added.

#### Chevron group surgical procedure

The technique adhered to the classic description by Schneider et al. [4]. A medial longitudinal incision was followed by a linear capsulotomy. To standardize soft-tissue balancing across both groups, an identical intra-articular lateral soft-tissue release (lateral capsule, adductor hallucis tendon, and lateral sesamoid ligament) was performed in all Chevron cases. A 60° V-shaped osteotomy was then created in the distal metaphyseal region of the first metatarsal. The capital fragment was laterally translated by approximately one-third of the metatarsal width (2–3 mm). Fixation was achieved with a headless compression screw, followed by a medial capsular reefing closure. As in the Modified group, no additional Akin or other phalangeal osteotomy was performed.

### Postoperative protocol

A uniform postoperative rehabilitation protocol was applied to all patients in both cohorts to minimize its potential influence on outcomes. Patients were mobilized immediately in a rigid-soled postoperative shoe, with weight-bearing restricted to the heel. No additional splints or braces were used. Active and passive range-of-motion exercises for the first metatarsophalangeal joint were initiated on the first postoperative day. Patients were routinely discharged on postoperative day 1. Pharmacological thromboprophylaxis with low-molecular-weight heparin (enoxaparin, 4,000 IU once daily) was prescribed for 28 days. Transition to full weight-bearing in regular footwear was permitted only after radiographic confirmation of osteotomy union, approximately 6–8 weeks postoperatively.

**Fig. 4:**
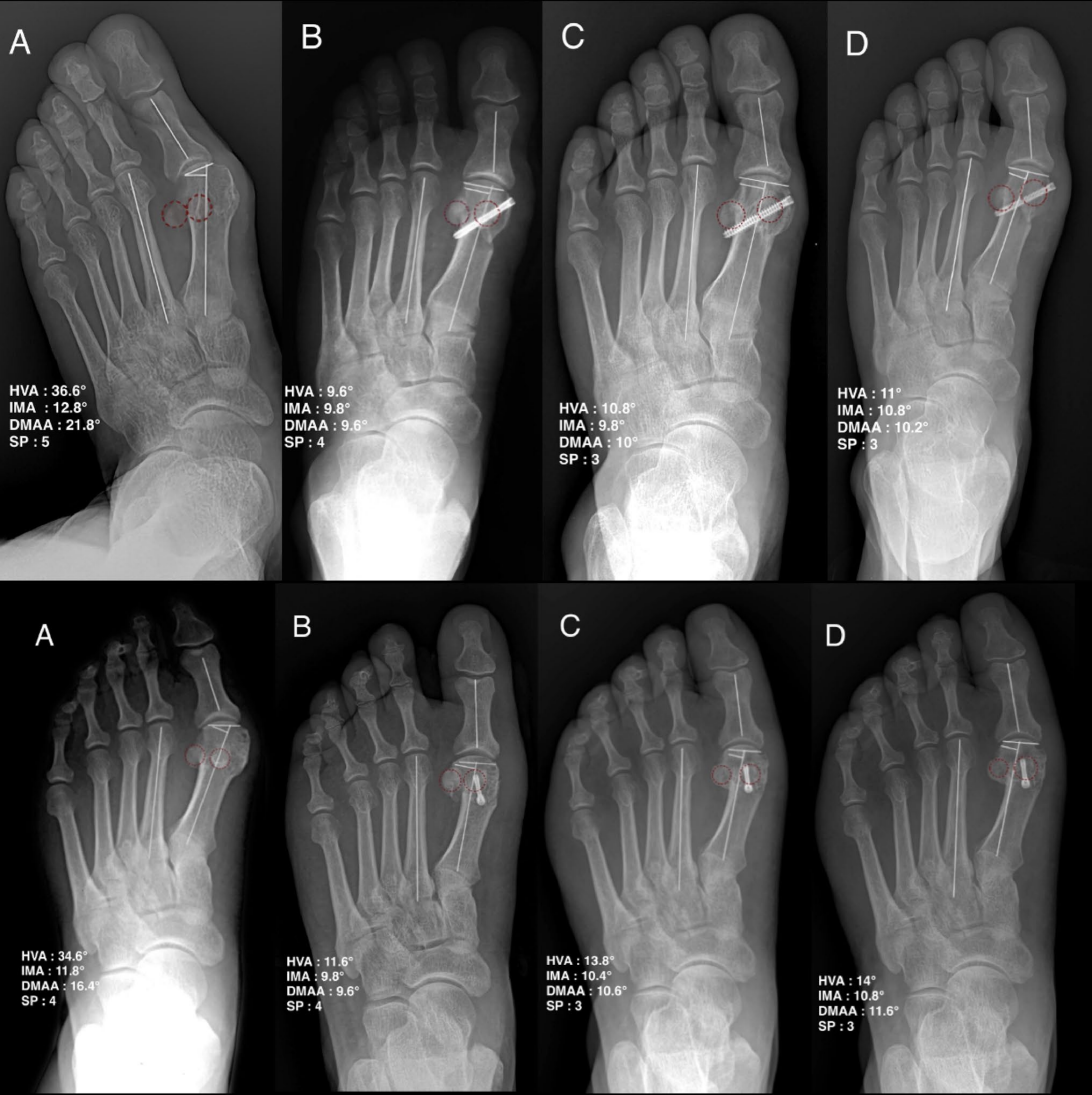
Baseline-matched weight-bearing anteroposterior (AP) radiographs (non-recurrent): Modified (upper) and Chevron (lower). **A** preoperative, **B** 6-8 weeks, **C** 1 year; **D** final. HVA/IMA/DMAA/sesamoid position annotated; red circles indicate the sesamoid complex.

**Fig. 5:**
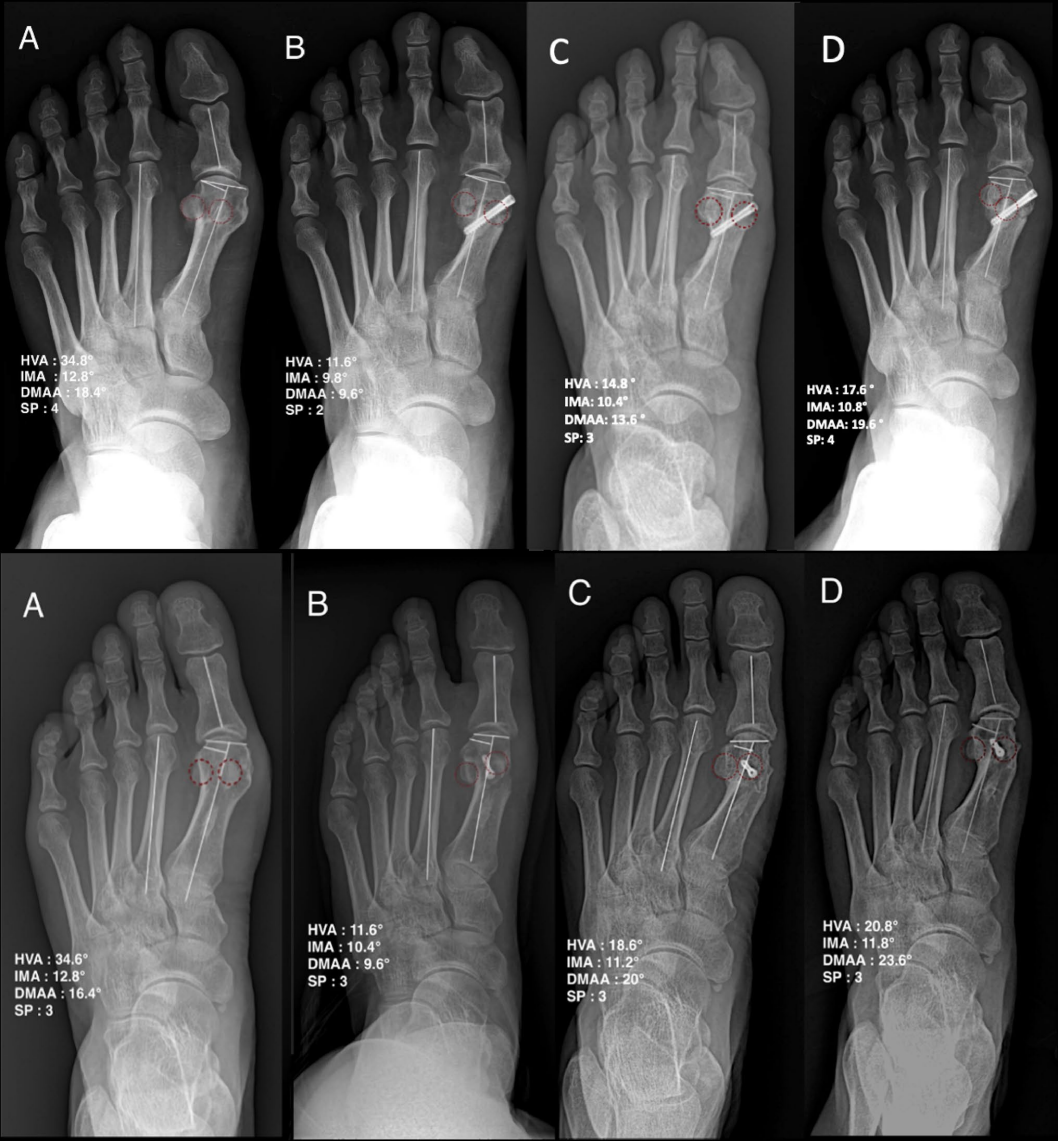
Baseline-matched weight-bearing AP radiographs (recurrent; final HVA > 15°), same layout/time points as Fig. 4. Overall recurrence: 4.3% (Modified) vs 27.8% (Chevron). Red circles indicate the sesamoid complex.

Radiographic evaluation was conducted using standardized weight-bearing anteroposterior and lateral foot radiographs retrieved from the institutional Picture Archiving and Communication System (PACS) (Figs. 4 and 5). Key alignment parameters, including the hallux valgus angle (HVA), intermetatarsal angle (IMA), distal metatarsal articular angle (DMAA), and medial sesamoid position, were measured at four time points: preoperative; first postoperative weight-bearing radiographs (6–8 weeks); 1-year postoperative; and final follow-up. Although DMAA was measured on standard anteroposterior radiographs, its interobserver reliability is limited and sensitive to X-ray beam inclination; values were therefore interpreted cautiously [14]. The medial sesamoid position was assessed using the seven-grade classification system described by Hardy-Clapham [15]. In this system, the position of the tibial sesamoid is evaluated relative to the mid-axial line of the first metatarsal shaft on the anteroposterior radiograph. Grades range from 1 (sesamoid completely medial to the axis) to 7 (complete lateral dislocation), with Grade 4 representing a centered position. To minimize potential measurement bias, all assessments were performed by an independent senior orthopedic surgeon who was not involved in the index procedures. Although the inherent radiographic differences between osteotomy configurations precluded blinding to the specific surgical technique, the assessor was strictly blinded to all patient identifiers, clinical outcomes (AOFAS/VAS scores), and complication status to mitigate detection bias. To minimize measurement error and improve data consistency, all radiographic parameters were independently assessed in two separate sessions with a minimum two-week interval by a single blinded assessor. The mean values from these two sessions were utilized for the final statistical analysis.

Clinical outcomes were assessed using the American Orthopaedic Foot & Ankle Society (AOFAS) Hallux-Metatarsophalangeal-Interphalangeal scale and the Visual Analog Scale (VAS) for pain (0–10). Preoperative AOFAS and VAS scores were obtained retrospectively from the medical records. Final follow-up scores were prospectively collected during the dedicated study visit. Any postoperative complications, including superficial or deep infection, wound-healing problems, hardware irritation, nonunion, transfer metatarsalgia, and radiographic signs of avascular necrosis, were systematically recorded.

**Fig. 6:**
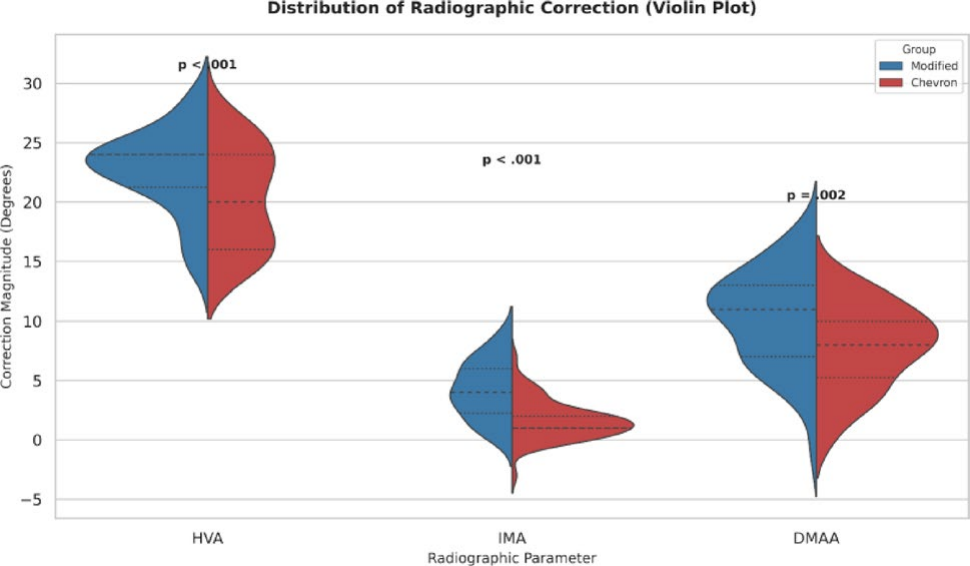
Distribution of radiographic correction magnitudes (change scores, Δ) for HVA, IMA, and DMAA. Violin plots comparing the Modified and Chevron groups. Change scores for radiographic angles were defined as ΔHVA/ΔIMA/ΔDMAA = preoperative - final (positive values indicate angular correction)

**Fig. 7:**
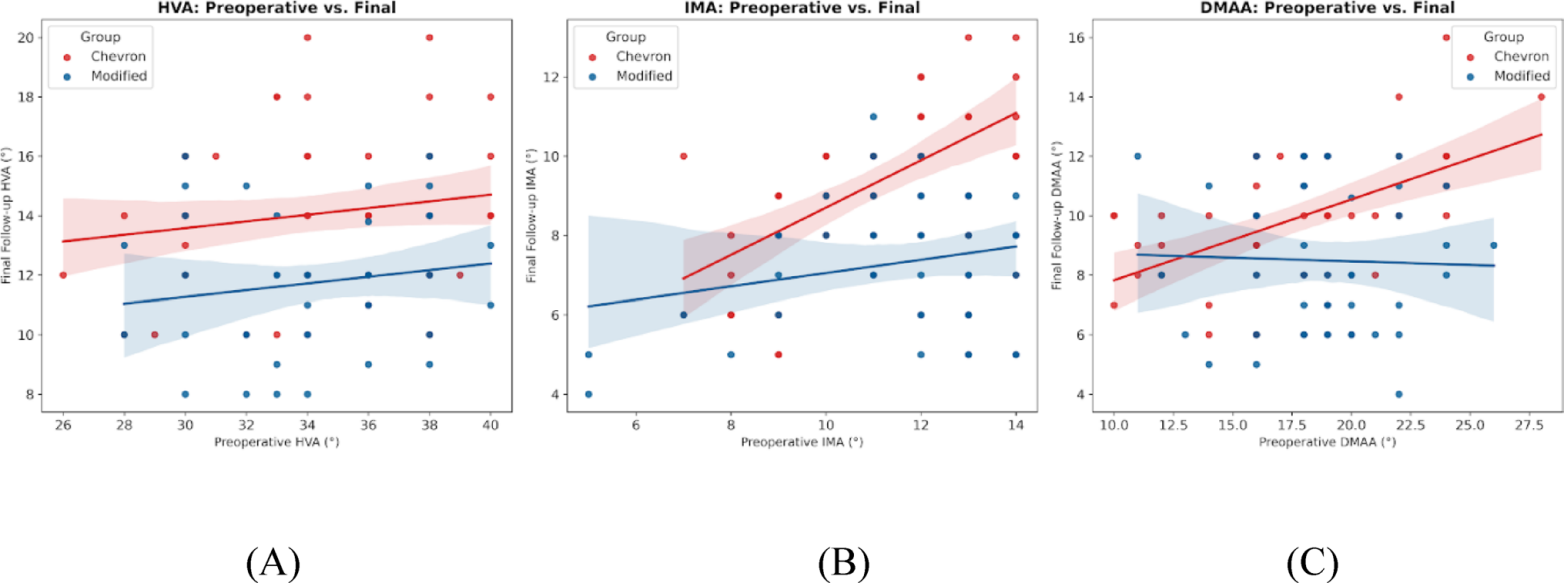
Scatter plots illustrating the relationship between preoperative and final follow-up radiographic angular measurements. The panels demonstrate the changes in **A** hallux valgus angle (HVA), **B** intermetatarsal angle (IMA), and **C** distal metatarsal articular angle (DMAA) for both the Chevron (red) and Modified (blue) osteotomy groups. Each solid dot represents an individual patient’s measurement. The solid lines depict the linear regression (line of best fit) for each respective group, while the shaded regions indicate the 95% confidence intervals

### Statistical analysis

Statistical analyses were performed using IBM SPSS Statistics for Windows, version 30.0 (IBM Corp., Armonk, NY, USA). Continuous variables (age, BMI, follow-up duration, radiographic angles, AOFAS scores, metatarsal shortening, and change scores [Δ]) were summarized as mean ± standard deviation (SD), ordinal variables (Hardy-Clapham medial sesamoid grades and VAS pain scores) as median [interquartile range, IQR], and categorical variables as counts and percentages. Analyses were conducted as complete-case analyses; missing values were not imputed. Normality of continuous variables and of paired differences (preoperative vs. final follow-up) was assessed using the Shapiro-Wilk test. Between-group comparisons of continuous outcomes were performed using Welch’s t-test, and within-group changes from preoperative to final follow-up in continuous outcomes (HVA, IMA, DMAA, and AOFAS score) were analyzed using two-tailed paired t-tests. Medial sesamoid position (Hardy-Clapham grades 1–7) and VAS pain scores (0–10) were treated as ordinal variables: between-group comparisons at each time point and of change scores (Δ) used the Mann-Whitney U test, and within-group changes over time used the Wilcoxon signed-rank test. Change scores (Δ) were defined as follows: ΔHVA, ΔIMA, and ΔDMAA = preoperative - final (positive values indicate angular correction); ΔAOFAS = final - preoperative (functional improvement); ΔVAS and Δsesamoid position = preoperative - final (pain reduction or sesamoid reduction). Categorical variables (sex, side, anesthesia type, recurrence status, and complication rates) were compared between groups using the χ² test or Fisher’s exact test, as appropriate. Radiographic recurrence was defined a priori as the primary endpoint (final HVA > 15°). As a sensitivity analysis to address potential confounding by indication due to baseline imbalances (notably preoperative medial sesamoid position), inverse probability of treatment weighting (IPTW) was performed. Propensity scores were estimated using logistic regression including age, sex, BMI, follow-up duration, and preoperative HVA, IMA, DMAA, and medial sesamoid position; stabilized weights were applied. An IPTW-weighted logistic regression model with radiographic recurrence as the dependent variable and treatment group as the independent variable was used to estimate the adjusted odds ratio (aOR) and 95% confidence interval (CI). Covariate balance before and after weighting was assessed using standardized mean differences (SMD), with |SMD| < 0.10 considered acceptable; balance diagnostics are reported in Supplementary Table S1. As a multiplicity sensitivity analysis, the Benjamini-Hochberg false discovery rate (BH-FDR) procedure was applied across the prespecified set of secondary between-group comparisons at final follow-up and their corresponding change scores (Δ) for HVA, IMA, DMAA, medial sesamoid position, AOFAS, and VAS (*n* = 12); BH-FDR-adjusted q-values are reported in Supplementary Table S2 (FDR = 0.05). All tests were two-sided, and *p* < 0.05 was considered statistically significant. In the tables, * denotes paired t-test, ** Welch’s t-test, † Mann-Whitney U test or Wilcoxon signed-rank test, and ‡ χ² test or Fisher’s exact test.

## Results

### Demographics and baseline characteristics

A total of 100 feet were analyzed, 46 in the modified distal metatarsal osteotomy group (Modified, Group 1) and 54 in the Chevron group (Group 2). Baseline demographics and perioperative characteristics (age, BMI, sex, operated side, type of anesthesia, and follow-up duration) were comparable between groups (all *p* > 0.05; Table 1). Preoperative HVA, IMA, DMAA, and AOFAS scores were also similar. In contrast, the Modified group exhibited a more lateralized medial sesamoid position at baseline, indicating slightly more advanced deformity (*p* < 0.001; Table 2). IPTW achieved acceptable balance across prespecified covariates (all weighted |SMD| < 0.10; Supplementary Table S1).

### Radiographic outcomes

Both procedures resulted in significant improvements in HVA, IMA, and DMAA from preoperative to final follow-up (all within-group *p* < 0.001; Table 3). However, the magnitude of correction consistently favored the modified osteotomy. Change scores (Δ) for HVA, IMA, and DMAA were all greater in the Modified group than in the Chevron group (all *p* ≤ 0.004; Table 3) (Fig. 6), and the Modified group maintained more favorable HVA and IMA values at all postoperative assessments (all *p* < 0.001; Table 2). For DMAA, the between-group difference did not reach statistical significance at the first postoperative weight-bearing assessment (6–8 weeks; *p* = 0.078), but favored the Modified group at 1-year postoperative (*p* = 0.003) and final follow-up (*p* = 0.002) (Table 2).

Radiographic drift between the first postoperative weight-bearing assessment (6–8 weeks) and final follow-up, derived from mean values in Table 2, was smaller in the Modified group (HVA + 0.4°; IMA + 0.0°) than in the Chevron group (HVA + 1.1°; IMA + 0.2°). DMAA remained stable in the Modified group (-0.4°) and was largely unchanged in the Chevron group (+ 0.1°), consistent with less loss of correction over time in the Modified cohort (Fig. 7).

**Fig. 8:**
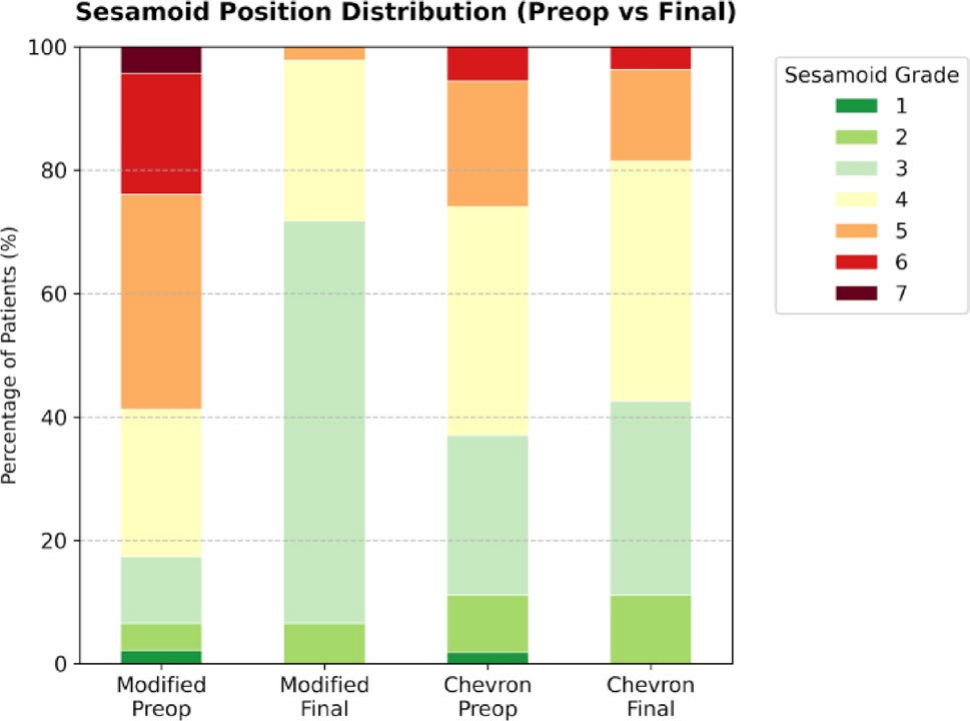
Sesamoid position distribution at baseline and final follow-up. Stacked bar chart demonstrating the percentage distribution of sesamoid position grades preoperatively and at the final follow-up. The comparative distribution is shown for both the Modified and Chevron osteotomy groups. The ordinal categorical data of sesamoid positions (grades 1 to 7) are represented by a color gradient ranging from normal alignment (grade 1, dark green) to severe subluxation (grade 7, dark red)

Despite starting with a worse medial sesamoid position, the Modified group achieved a markedly larger improvement in sesamoid grade than the Chevron group (Δsesamoid position, *p* < 0.001) and a more central final sesamoid position (*p* = 0.010; Tables 2 and 3) (Fig. 8). Metatarsal shortening at final follow-up was modest and similar between groups (*p* = 0.145; Table 2).

### Clinical outcomes

Clinical outcomes improved substantially in both cohorts. AOFAS scores increased by approximately 30 points in each group, reaching similarly high values at final follow-up; neither the final AOFAS scores nor the magnitude of improvement differed significantly between techniques (*p* = 0.621 and *p* = .157, respectively; Tables 2 and 3). VAS pain scores decreased from severe preoperative levels to low values at final follow-up in both groups. The magnitude of pain reduction (ΔVAS) was comparable between groups (*p* = 0.104), although the distribution of final VAS scores showed slightly less residual pain in the Modified group (*p* = 0.043; Tables 2 and 3). In BH-FDR sensitivity analysis across prespecified secondary endpoints, the between-group difference in final VAS did not meet q < 0.05 (q = 0.057; Supplementary Table S2).

### Recurrence

Radiographic recurrence, defined as a final HVA > 15°, was significantly less frequent after the modified osteotomy than after the Chevron procedure (4.3% vs. 27.8%; absolute risk difference, -23.4%; relative risk 0.16, 95% CI 0.04–0.65; *p* = 0.003; Table 4). When a stricter definition was applied (final HVA ≥ 20°), severe recurrence was rare and did not differ significantly between groups (0% vs. 3.7%; *p* = 0.498; Table 4). In the IPTW-weighted analysis adjusting for baseline imbalances, the modified technique was associated with lower odds of radiographic recurrence (aOR 0.09, 95% CI 0.01–0.60; *p* = 0.013; Supplementary Table S3).

In a post-hoc exploratory analysis, final functional scores did not differ significantly between patients with and without radiographic recurrence (AOFAS: with recurrence 96.8 ± 3.5 vs. without 94.9 ± 5.7, *p* = 0.093; VAS: median 1.0 [0.0–2.0] vs. 1.0 [0.0–2.0], *p* = 0.896), although this comparison should be interpreted with caution. The limited number of recurrent cases (*n* = 17) renders this post-hoc subgroup analysis inherently underpowered to definitively exclude a functional decrement.

### Complications

No cases of nonunion or avascular necrosis were observed in either group. Rates of superficial infection and hardware removal were low and similar between techniques (infection, 2.2% vs. 3.7%; hardware removal, 4.3% vs. 5.6% in the Modified and Chevron groups, respectively), with no statistically significant between-group differences (all *p* = 1.000; Table 5).

**Table 1:** Demographic and baseline characteristics of the Modified and Chevron groups

Parameter	Modified group (*n* = 46)	Chevron group (*n* = 54)	*p* value
Age (years)	40.0 ± 9.5	39.8 ± 9.0	*p* = 0.904**
Gender (female/male),* n*	38 / 8	50 / 4	*p* = 0.222‡
Side (right/left),* n*	25 / 21	26 / 28	*p* = 0.676‡
BMI (kg/m²)	30.2 ± 2.4	30.0 ± 2.2	*p* = 0.664**
Type of anesthesia (spinal/general),* n*	39 / 7	44 / 10	*p* = 0.864‡
Follow-up (months)	101.2 ± 11.2	101.2 ± 11.8	*p* = 0.969**

Values are presented as mean ± standard deviation (SD) for continuous variables and as counts for categorical variables. *P* values were obtained using Welch’s t-test for continuous variables (**) and the χ² test or Fisher’s exact test, as appropriate, for categorical variables (‡)

**Table 2:** Radiographic outcomes at four time points and clinical outcomes at baseline and final follow-up in the modified and Chevron groups

Parameter	Time point	Modified group (*n* = 46)	Chevron group (*n* = 54)	*p* value (between groups)
HVA (°)	Preoperative	34.4 ± 3.1	34.4 ± 3.9	*p* = 0.957**
	Postoperative	11.4 ± 2.3	13.0 ± 2.0	*p* < 0.001**
	1-year postoperative	11.8 ± 2.2	13.7 ± 2.2	*p* < 0.001**
	Final follow-up	11.8 ± 2.2	14.1 ± 2.4	*p* < 0.001**
IMA (°)	Preoperative	11.5 ± 2.2	10.9 ± 2.0	*p* = 0.128**
	Postoperative	7.3 ± 1.6	9.0 ± 1.9	*p* < 0.001**
	1-year postoperative	7.3 ± 1.6	9.2 ± 1.9	*p* < 0.001**
	Final follow-up	7.3 ± 1.6	9.2 ± 1.9	*p* < 0.001**
DMAA (°)	Preoperative	18.8 ± 3.2	17.8 ± 4.2	*p* = 0.196**
	Postoperative	8.9 ± 2.6	9.8 ± 1.9	*p* = 0.078**
	1-year postoperative	8.5 ± 2.4	9.9 ± 2.0	*p* = 0.003**
	Final follow-up	8.5 ± 2.4	9.9 ± 2.1	*p* = 0.002**
Medial sesamoid position (Hardy-Clapham 1–7)	Preoperative	5.0 [4.0–5.0] (1–7)	4.0 [3.0-4.8] (1–6)	*p* < 0.001†
	Postoperative	3.0 [3.0–4.0] (2–5)	4.0 [3.0–4.0] (2–6)	*p* = 0.411†
	1-year postoperative	3.0 [3.0–4.0] (2–5)	4.0 [3.0–4.0] (2–6)	*p* = 0.018†
	Final follow-up	3.0 [3.0–4.0] (2–5)	4.0 [3.0–4.0] (2–6)	*p* = 0.010†
AOFAS score (points)	Preoperative	66.7 ± 9.2	69.1 ± 10.1	*p* = 0.235**
	Final follow-up	95.5 ± 5.5	95.0 ± 5.4	*p* = 0.621**
Metatarsal shortening (mm)	Final follow-up	0.8 ± 0.8	1.1 ± 0.9	*p* = 0.145**
VAS pain score (0–10)	Preoperative	9.0 [8.0–9.0]	9.0 [8.0–9.0]	*p* = 0.945†
	Final follow-up	1.0 [0.0–1.0]	1.0 [1.0–2.0]	*p* = 0.043†

HVA, IMA, DMAA, AOFAS, and metatarsal shortening are mean ± SD; VAS and sesamoid position are median [IQR] (sesamoid: min-max). Postoperative refers to the first postoperative weight-bearing radiographs (6-8 weeks)

** Welch’s t-test; † Mann-Whitney U test

**Table 3:** Within-group changes and between-group comparisons of radiographic and clinical outcomes (Δ)

Parameter	Δ Modified group (*n* = 46)	*p* value (within Modified)	Δ Chevron group (*n* = 54)	*p* value (within Chevron)	*p* value (between Δ)
Δ HVA (°)	22.6 ± 3.5	*p* < .001*	20.3 ± 4.2	*p* < .001*	*p* = 0.004**
Δ IMA (°)	4.2 ± 2.4	*p* < .001*	1.6 ± 1.7	*p* < .001*	*p* < 0.001**
Δ DMAA (°)	10.3 ± 4.0	*p* < .001*	7.9 ± 3.5	*p* < .001*	*p* = 0.002**
Δ medial sesamoid position	2.0 [1.0–2.0]	*p* < .001†	0.0 [0.0–0.0]	*p* = .144†	*p* < 0.001†
Δ AOFAS score (points)	28.8 ± 7.5	*p* < .001*	25.9 ± 12.3	*p* < .001*	*p* = 0.157**
Δ VAS pain score (0–10)	8.0 [7.0–8.0]	*p* < .001†	7.5 [6.3-8.0]	*p* < .001†	*p* = 0.104†

Δ HVA, Δ IMA, and Δ DMAA were calculated as preoperative - final values (positive values indicate angular correction). Δ AOFAS was calculated as final - preoperative (functional improvement). Δ VAS pain score and Δ medial sesamoid position were calculated as preoperative - final (positive values indicate pain reduction or sesamoid reduction). Values are presented as mean ± SD for continuous variables and as median [IQR] for ordinal variables. Within-group comparisons were performed using paired t-tests for continuous variables (*) and Wilcoxon signed-rank tests for ordinal variables (†). Between-group comparisons of Δ were performed using Welch’s t-test for continuous variables (**) and the Mann-Whitney U test for ordinal variables (†)

**Table 4:** Comparison of recurrence rates between Modified and Chevron groups

Outcome	Group 1 (Modified) (*n* = 46)	Group 2 (Chevron) (*n* = 54)	*p* value‡
Recurrence rate (Final HVA > 15°)	2 (4.3%)	15 (27.8%)	*p* = 0.003
Severe recurrence (Final HVA ≥ 20°)	0 (0.0%)	2 (3.7%)	*p* = 0.498

Values are n (%). Recurrence was defined as a final hallux valgus angle (HVA) > 15° at the last follow-up, and severe recurrence as final HVA ≥ 20°

‡ P values were calculated using Fisher’s exact test

**Table 5:** Comparison of complications between Modified and Chevron groups

Complication / Outcome	Group 1 (Modified) *n* (%)	Group 2 (Chevron) *n* (%)	*p* value‡
Nonunion	0 (0.0%)	0 (0.0%)	*p* = 1.000
Avascular necrosis	0 (0.0%)	0 (0.0%)	*p* = 1.000
Infection	1 (2.2%)	2 (3.7%)	*p* = 1.000
Hardware removal	2 (4.3%)	3 (5.6%)	*p* = 1.000

Values are n (%)

‡ P values were calculated using Fisher’s exact test

## Discussion

The principal finding of this study is that the modified rotational wedge distal first metatarsal osteotomy was associated with more durable radiographic correction than the conventional distal Chevron osteotomy at a mean follow-up of 101.2 months (approximately 8.4 years). Compared with the Chevron group, the Modified group demonstrated lower final HVA, IMA, and DMAA values, improved sesamoid reduction, and a lower rate of radiographic recurrence (final HVA > 15°). Because soft-tissue balancing was standardized across both cohorts through an identical intra-articular lateral release, the observed between-group differences most plausibly reflect the osteotomy geometry itself and its capacity for multiplanar correction. The Modified group exhibited greater baseline sesamoid displacement, raising concern for confounding by indication and the possibility that the two cohorts represent different deformity phenotypes; however, an IPTW sensitivity analysis preserved the direction and magnitude of the recurrence effect, supporting the robustness of the primary comparison. Because coronal rotation was not quantified directly, sesamoid position was interpreted as a radiographic surrogate for transverse-plane realignment, an approach that has been validated in the absence of three-dimensional imaging [16]. 

Distal Chevron osteotomy remains well-established for mild-to-moderate hallux valgus [4, 5], but progressive loss of correction has been documented, particularly with incomplete sesamoid reduction or elevated DMAA [6, 7]. In the present cohort, both techniques produced significant improvement; however, the modified osteotomy maintained correction more effectively over nearly a decade. Our results support postoperative sesamoid position as an important determinant of long-term durability, though the evidence is not entirely uniform [11, 12], and suggest that incomplete sesamoid reduction may contribute to recurrence rather than representing a purely radiographic epiphenomenon [17, 18]. Postoperative sesamoid position has also been linked to functional outcome and patient satisfaction, reinforcing its relevance beyond radiographic assessment [17]. Despite more lateralized baseline sesamoids, the Modified group achieved a more central final position, consistent with more effective three-dimensional correction when combined with fluoroscopy-guided intraoperative derotation [16, 19]. Notably, lateral soft-tissue release alone has not been associated with durable correction in the percutaneous setting [20]. 

Biomechanical testing has demonstrated comparable bending stiffness and load-to-failure properties between a newly defined distal osteotomy and distal Chevron osteotomy [21], and metatarsal shortening was modest and similar between groups in the present cohort. Favorable early outcomes reported with other contemporary distal three-dimensional constructs further support the structural feasibility of multiplanar correction at the distal first metatarsal [22]. Similarly, minimally invasive adaptations of the Chevron osteotomy have demonstrated reliable intermetatarsal correction, highlighting the ongoing clinical evolution of these distal techniques [23]. 

Radiographic superiority did not translate into superior patient-reported outcomes. This dissociation reinforces the principle that improved radiographic alignment does not necessarily equate to superior function. Final AOFAS results were excellent and statistically comparable in both groups, consistent with prior reports demonstrating weak correlations between radiographic alignment and AOFAS scores and ceiling effects that limit discriminative capacity at long-term follow-up [24, 25]. Although final VAS pain scores favored the Modified group in unadjusted analysis, this difference was not retained after Benjamini-Hochberg false discovery rate correction (q = 0.057), underscoring the need for cautious interpretation of secondary endpoints. Moreover, recurrence was defined by a radiographic threshold (final HVA > 15°) rather than by patient-perceived failure, need for reoperation, or time-to-event analysis; severe recurrence (final HVA ≥ 20°) was uncommon and did not differ significantly between groups. Consistent with this interpretation, post-hoc analysis revealed no significant difference in final AOFAS or VAS scores between patients with and without radiographic recurrence, likely reflecting the predominantly mild recurrences in this cohort, though the high risk of Type II error due to the small sample size of the recurrence group (*n* = 17) precludes definitive conclusions regarding functional impact. The clinical significance of radiographic durability should therefore be interpreted conservatively and validated against patient-centered outcomes in future studies.

Several limitations should be considered when interpreting these findings. The retrospective, nonrandomized, single-center design (two surgeons) introduces selection bias risk and limits external validity, and residual confounding cannot be excluded despite IPTW adjustment for measured covariates. Unmeasured confounders, including temporal trends in surgical practice, may have influenced group allocation and outcomes. DMAA measurement on anteroposterior radiographs has well-documented reliability limitations, and between-group differences in this parameter should be interpreted with caution [14]. Radiographic assessment was limited to conventional weight-bearing plain radiographs; weight-bearing computed tomography would permit more direct quantification of first metatarsal pronation and three-dimensional correction [26]. Formal inter- and intra-observer reliability statistics were not calculated, although all measurements were performed twice in a standardized fashion by a single blinded assessor and mean values were used. Although an a priori sample size calculation was not performed, a post-hoc analysis requested during peer review—based on the large absolute risk difference (23.4%) in our primary endpoint—yielded > 90% power, justifying the cohort size for radiographic recurrence. However, the study remains inherently underpowered to definitively assess differences in severe recurrence rates, subtle functional disparities, or rare complications. Survivorship analyses based on revision surgery and patient-reported dissatisfaction were not incorporated into the definition of recurrence.

Despite these limitations, the well-characterized cohort, standardized soft-tissue balancing, and a mean follow-up exceeding 8 years provide clinically meaningful comparative data. Within this context, the modified rotational wedge osteotomy was associated with more durable radiographic correction and a lower radiographic recurrence rate than the distal Chevron osteotomy, without higher complication rates. These findings should be regarded as hypothesis-generating. Prospective, multicenter, randomized trials incorporating three-dimensional imaging and validated patient-centered outcome measures are warranted to confirm these results and to clarify the clinical significance of radiographic durability.

## Conclusion

In this long-term retrospective cohort study, the modified rotational wedge osteotomy was associated with more durable radiographic correction, improved sesamoid reduction, and a lower rate of radiographic recurrence than distal Chevron osteotomy; this association persisted after IPTW adjustment for baseline imbalances. Clinical outcomes improved substantially and were comparable between groups, and complications were uncommon. Given the retrospective, nonrandomized design and the absence of superiority in patient-reported outcomes, these findings should be regarded as hypothesis-generating and interpreted with appropriate caution. Prospective, multicenter, randomized trials incorporating three-dimensional imaging and validated patient-reported outcomes are warranted to confirm these results and to determine whether the observed radiographic advantages translate into meaningful clinical benefit.

## Supplementary Information

Below is the link to the electronic supplementary material.

Supplementary Material 1

Supplementary Material 2

Supplementary Material 3

## Data Availability

The datasets generated during and/or analysed during the current study are available from the corresponding author on reasonable request.

## Abbreviations

AOFAS: American Orthopaedic Foot & Ankle Society
aOR: Adjusted odds ratio
AP: Anteroposterior
BH-FDR: Benjamini–Hochberg false discovery rate
BMI: Body mass index
CI: Confidence interval
DMAA: Distal metatarsal articular angle
HVA: Hallux valgus angle
IMA: Intermetatarsal angle
IPTW: Inverse probability of treatment weighting
IQR: Interquartile range
IU: International units
PACS: Picture archiving and communication system
SD: Standard deviation
SMD: Standardized mean difference
VAS: Visual analog scale
