# Correction: Modified rotational wedge distal metatarsal osteotomy versus chevron osteotomy for hallux valgus: long-term radiographic and clinical outcomes

**DOI:** 10.1007/s00402-026-06420-2

**Published:** 2026-07-17

**Authors:** Ahmet Aybar, Kemal Gokkus, Erdem Özden, Mehmet Hamdi Çetin, Mehmet Ümit Çetin

**Affiliations:** 1 Department of Orthopedics and Traumatology, University of Health Sciences, Gaziosmanpasa Training and Research Hospital, Kemal Gokkus, Istanbul, Turkey; 2 Department of Orthopedics and Traumatology, Alanya Research and Practice Center, Baskent University School of Medicine, Erdem Özden, Antalya, Turkey; 3 Department of Orthopedics and Traumatology, Faculty of Medicine, Tekirdag Namik Kemal University, Tekirdag, Turkey

**Correction: Archives of Orthopaedic and Trauma Surgery (2026) 146:149** 10.1007/s00402-026-06315-2

In this article, the ethics approval statement in the Materials and Methods section was incorrectly given as

The protocol was approved by the Institutional Review Board (Approval No. Blinded for Review) and complied with the Declaration of Helsinki.

but should have been

The protocol was approved by the Clinical Research Ethics Committee of Gaziosmanpasa Training and Research Hospital (Decision No. 86, dated 25 May 2022) and complied with the Declaration of Helsinki.

The original article has been corrected.

