# Supplementary material for: Modified rotational wedge distal metatarsal osteotomy versus chevron osteotomy for hallux valgus: long-term radiographic and clinical outcomes
Source: Arch Orthop Trauma Surg. 2026 Apr 17;146(1):149. doi: 10.1007/s00402-026-06315-2 (PMC13090300; doi:10.1007/s00402-026-06315-2)
Supplement: Supplementary file 1 — Supplementary Material 1 [file 402_2026_6315_MOESM1_ESM.docx]

**Table S1. Baseline covariate balance before and after inverse probability of treatment weighting (IPTW).**

| **Covariate** | **\|SMD\| (unweighted)** | **\|SMD\| (IPTW-weighted)** |
| --- | --- | --- |
| **Age (years)** | 0.02 | 0.05 |
| **Female sex** | 0.30 | 0.09 |
| **BMI (kg/m²)** | 0.09 | 0.06 |
| **Follow-up (months)** | 0.01 | 0.02 |
| **Preoperative HVA (°)** | 0.01 | 0.00 |
| **Preoperative IMA (°)** | 0.31 | 0.02 |
| **Preoperative DMAA (°)** | 0.26 | 0.08 |
| **Preoperative medial sesamoid position** | 0.66 | 0.02 |

Abbreviations: IPTW, inverse probability of treatment weighting; SMD, standardized mean difference. Lower values indicate better balance; values <0.10 are commonly considered acceptable.
