# Supplementary material for: Modified rotational wedge distal metatarsal osteotomy versus chevron osteotomy for hallux valgus: long-term radiographic and clinical outcomes
Source: Arch Orthop Trauma Surg. 2026 Apr 17;146(1):149. doi: 10.1007/s00402-026-06315-2 (PMC13090300; doi:10.1007/s00402-026-06315-2)
Supplement: Supplementary file 2 — Supplementary Material 2 [file 402_2026_6315_MOESM2_ESM.docx]

**Table S2. False discovery rate (Benjamini–Hochberg) sensitivity analysis for prespecified secondary between-group comparisons.**

| **Endpoint** | **Test** | **p value** | **BH-FDR q value** |
| --- | --- | --- | --- |
| **Final HVA (°)** | Welch’s t-test | <0.001 | <0.001 |
| **Final IMA (°)** | Welch’s t-test | <0.001 | <0.001 |
| **Final DMAA (°)** | Welch’s t-test | .002 | .004 |
| **Final medial sesamoid position (Hardy–Clapham 1–7)** | Mann–Whitney U | .010 | .014 |
| **Final AOFAS score (points)** | Welch’s t-test | .621 | .621 |
| **Final VAS pain score (0–10)** | Mann–Whitney U | .043 | .057 |
| **ΔHVA (preoperative − final)** | Welch’s t-test | .004 | .007 |
| **ΔIMA (preoperative − final)** | Welch’s t-test | <0.001 | <0.001 |
| **ΔDMAA (preoperative − final)** | Welch’s t-test | .002 | .004 |
| **ΔMedial sesamoid position (preoperative − final)** | Mann–Whitney U | <0.001 | <0.001 |
| **ΔAOFAS score (final − preoperative)** | Welch’s t-test | .157 | .171 |
| **ΔVAS pain score (preoperative − final)** | Mann–Whitney U | .104 | .125 |

Notes: This sensitivity analysis was performed across the prespecified set of secondary endpoints shown below. The primary endpoint (radiographic recurrence at final follow-up) was not included in this correction. FDR was controlled at 0.05.
