# Supplementary material for: Modified rotational wedge distal metatarsal osteotomy versus chevron osteotomy for hallux valgus: long-term radiographic and clinical outcomes
Source: Arch Orthop Trauma Surg. 2026 Apr 17;146(1):149. doi: 10.1007/s00402-026-06315-2 (PMC13090300; doi:10.1007/s00402-026-06315-2)
Supplement: Supplementary file 3 — Supplementary Material 3 [file 402_2026_6315_MOESM3_ESM.docx]

**Table S3. Treatment effect estimates for the primary endpoint (radiographic recurrence at final follow-up).**

| **Measure** | **Estimate** | **95% CI** | **p value** |
| --- | --- | --- | --- |
| **Event rate (Modified vs Chevron)** | 2/46 (4.3%) vs 15/54 (27.8%) | — | — |
| **Unadjusted relative risk (RR)** | 0.16 | 0.04–0.65 | .003 |
| **IPTW-adjusted odds ratio (OR)** | 0.09 | 0.01–0.60 | .013 |

Recurrence was defined as final HVA > 15°. Unadjusted comparisons are based on observed event rates. The IPTW-adjusted estimate is derived from IPTW-weighted logistic regression with treatment group as the independent variable.
